# Diagnostic Performance of CBC-Derived Inflammatory Ratios (SII, NLR, PLR) for Identifying Biopsy-Proven Microscopic Gut Inflammation Despite Normal Endoscopy

**DOI:** 10.7759/cureus.116884

**Published:** 2026-09-24

**Authors:** Hamid Ahmad, Muhammad Muzammil Hussain, Aya Ibrihim, Saad Chaudry, Nitin Wathore, Abdul Rashid Sheikh, Tosin Ayantoyinbo, Noor-ul-Huda Tunio, Zukhruf Zahoor, Lamis Beshir, Ali Farid

**Affiliations:** 1 Internal Medicine, Mardan Medical Complex, Mardan, PAK; 2 Internal Medicine, Federal Government Polyclinic Hospital, Islamabad, PAK; 3 Dermatology, Ala-Too International University, Bishkek, KGZ; 4 General Physician, Islam Medical College, Sialkot, PAK; 5 Internal Medicine, Gulf Medical University, Dubai, ARE; 6 Internal Medicine, Shaikh Zayed Postgraduate Medical Institute, Lahore, PAK; 7 Internal medicine, BronxCare Health System, New York, USA; 8 Medicine, Liaquat University of Medical and Health Sciences, Jamshoro, PAK; 9 Medicine, Chaudhry Hospital, Lahore, PAK; 10 Medicine, Services Institute of Medical Sciences, Lahore, PAK; 11 Clinical Immunology, Sudan Medical Specialization Board, Khartoum, SDN; 12 Internal Medicine, Omdurman Military Hospital, Khartoum, SDN; 13 Internal Medicine, University of Health Sciences, Lahore, PAK

**Keywords:** biopsy, endoscopy, gastrointestinal diseases, inflammation, neutrophils

## Abstract

Background

Intestinal inflammation may be present in the early stages without endoscopic abnormalities, making timely diagnosis difficult. The inflammatory ratios calculated from complete blood count (CBC) have been proposed as noninvasive markers of inflammatory activity; among these, three are noteworthy: the neutrophil-to-lymphocyte ratio (NLR), the platelet-to-lymphocyte ratio (PLR), and the systemic immune-inflammation index (SII).

Methods

A cross-sectional analytical study was conducted among 350 adults undergoing upper gastrointestinal endoscopy or colonoscopy with biopsy. Patients were divided into two groups: Group A (patients with normal endoscopy and normal biopsy; n=106) and Group B (patients with normal endoscopy and biopsy-proven microscopic inflammation; n=244). Hematological parameters were obtained before the procedure and used to calculate CBC-derived inflammatory ratios. To compare inflammatory indices between groups, Mann-Whitney U tests were used. Binary logistic regression was used to evaluate associations between inflammatory indices and biopsy-proven microscopic gut inflammation after adjustment for selected clinical variables. Receiver operating characteristic (ROC) curve analysis was performed to assess the discriminatory performance of NLR, PLR, and SII.

Results

NLR, PLR, and SII were significantly elevated in those with biopsy-proven microscopic inflammation in the absence of endoscopic inflammation (all p<0.001). Multivariable logistic regression demonstrated significant associations between NLR (odds ratio (OR)=1.57, 95% confidence interval (CI): 1.340-1.835, p<0.001), PLR (OR=1.012, 95% CI: 1.004-1.020, p=0.003), and SII (OR=1.025, 95% CI: 1.019-1.031, p<0.001) and biopsy-proven microscopic gut inflammation. ROC analysis showed that SII had the highest discriminatory performance (area under the curve (AUC)=0.776, 95% CI: 0.720-0.831), followed by PLR (AUC=0.729, 95% CI: 0.668-0.790) and NLR (AUC=0.684, 95% CI: 0.620-0.748).

Conclusion

CBC-derived inflammatory ratios, especially SII, could serve as simple, inexpensive, and non-invasive adjunctive markers for identifying biopsy-proven microscopic gut inflammation despite normal endoscopic findings. Their use may provide additional information for identifying patients who may warrant further gastrointestinal evaluation.

## Introduction

Gastrointestinal inflammation is a key mechanism in the pathogenesis of many inflammatory gastrointestinal disorders, including chronic inflammatory gastrointestinal diseases, infectious enterocolitis, and inflammatory bowel disease (IBD) [1]. Clinically, early detection of intestinal inflammation is critical, as it can progress to mucosal injury and complications, thereby affecting quality of life [2,3].

Although endoscopy is the gold standard for evaluating gastrointestinal inflammation, it is invasive and costly and not feasible for routine early screening [4,5]. Moreover, inflammatory activity may be present but not yet visible on endoscopy, requiring simple, non-destructive markers that detect inflammation at an earlier stage [6].

The neutrophil-to-lymphocyte ratio (NLR), platelet-to-lymphocyte ratio (PLR), and systemic immune-inflammation index (SII) are potential inflammatory markers based on complete blood count (CBC) [7]. These markers are cheap, widely available, and frequently used in clinical practice [8]. Neutrophils, platelets, and lymphocytes may be elevated due to inflammatory activation, and lymphocyte levels may be reduced due to immune abnormalities and disrupted immune homeostasis [9].

SII, a composite index of neutrophils, platelets, and lymphocytes, may better capture the burden of inflammation than individual indices and may provide additional information for identifying patients with possible inflammatory activity. Researchers have investigated these markers as potential indicators of inflammatory activity in patients with no obvious endoscopic findings. However, evidence for these markers in detecting early gut inflammation in patients with no endoscopic changes remains limited [5,10].

This study is novel because it evaluates the diagnostic and discriminatory performance of CBC-derived inflammatory ratios specifically for identifying biopsy-proven microscopic gut inflammation despite normal endoscopic findings. In this study, we investigated the potential of SII, NLR, and PLR as easily measurable blood markers of gut inflammation, compared with previous studies that have focused primarily on established IBD or more advanced inflammatory states. Therefore, this study evaluated the association between inflammatory ratios and biopsy-proven microscopic gut inflammation, compared the inflammatory indices between participants with and without biopsy-proven microscopic inflammation despite normal endoscopic findings, and determined which index showed the strongest association and best discriminatory performance for biopsy-proven microscopic gut inflammation.

## Materials and methods

Study design

This cross-sectional analytical study was conducted in a hospital to evaluate the diagnostic/discriminatory performance of inflammatory ratios readily obtained from CBC analysis, such as SII, NLR, and PLR, as markers of biopsy-proven microscopic gut inflammation despite normal endoscopic findings. This study aimed to determine whether these hematological inflammatory indices were associated with biopsy-proven microscopic gut inflammation in patients with normal endoscopic findings.

Study setting and duration

The study was carried out in gastroenterology and endoscopy wards of tertiary care centers. Patients who visited the outpatient and inpatient departments for gastrointestinal complaints and underwent colonoscopy or upper gastrointestinal endoscopy were selected. Data collection was conducted for five months from February 2026 to June 2026.

Study population

Adult patients presenting with gastrointestinal symptoms suggestive of intestinal inflammation were scheduled for colonoscopy or upper gastrointestinal endoscopy and biopsy. Symptoms included abdominal pain, chronic diarrhea, bloating, alterations in bowel habits, or other clinical conditions that increased the likelihood of inflammatory gastrointestinal disease. The inclusion and exclusion criteria used for participant selection are presented in Table 1.

**Table 1:** Eligibility Criteria for Study Participants

Inclusion Criteria	Exclusion Criteria
Adults aged 18 years or older were eligible for inclusion.	Patients with active infection or sepsis were excluded.
Patients undergoing colonoscopy or upper gastrointestinal endoscopy for gastrointestinal symptoms were included.	Patients with hematological disorders or malignancy were excluded.
Patients who underwent biopsy during the endoscopic procedure were included.	Patients with autoimmune disease or pregnancy were excluded.
We included patients with a complete blood count (CBC) report obtained within 24 hours before the procedure.	Patients who had undergone recent surgery or experienced major trauma were excluded, and those receiving steroids or immunosuppressive drugs were also excluded.

Sampling technique

We used a non-probability consecutive sampling technique. Eligible patients were recruited sequentially during the study period according to the predefined inclusion and exclusion criteria. The sample size was determined using the WHO sample size calculator, based on a 95% confidence level, 80% power, and the expected frequency of microscopic inflammation reported in previous studies. We calculated a sample size of 318 participants. The final sample included 350 participants, allowing for approximately 10% incomplete data.

Data collection procedure

Patients* *were identified for colonoscopy or upper gastrointestinal (GI) endoscopy by checking the daily endoscopy lists. Individuals who fulfilled the inclusion criteria were approached before the procedure, and written informed consent was obtained. The participant demographic and clinical data collected were structured and included age, sex, smoking history, gastrointestinal symptoms, duration of symptoms, family history of gastrointestinal disease, history of digestive system disease, drug history, and history of digestive system examinations. We considered potential clinical conditions that could influence CBC-derived inflammatory indices during participant selection. We excluded patients with active infection or sepsis according to the predefined exclusion criteria. The CBC values obtained from the hospital laboratory database within 24 hours before the endoscopic procedure were used. Absolute neutrophil count (ANC), absolute lymphocyte count, and platelet count were recorded as hematological parameters. These parameters were then used to calculate the inflammatory ratios, which were subsequently analyzed in the study.

ANC and absolute lymphocyte count (ALC) were measured, and the NLR was calculated. NLR was calculated by dividing the absolute neutrophil count by the absolute lymphocyte count. PLR was calculated by dividing the platelet count by the absolute lymphocyte count. SII was calculated as platelet count × neutrophil count/absolute lymphocyte count.

Endoscopic findings were collected from official procedure reports prepared by consultant gastroenterologists. The results were divided into those corresponding to normal mucosa, mild erythema, edema, or ulceration. Patients with an endoscopically normal appearance were the main focus of the study because the analysis examined biopsy-proven microscopic inflammation despite the absence of visible mucosal abnormalities on endoscopy.

Biopsy samples obtained during upper gastrointestinal endoscopy or colonoscopy were submitted for routine histopathological examination. Histopathological reports were reviewed to determine whether microscopic inflammatory changes were present despite a normal endoscopic appearance. For the present analysis, microscopic gut inflammation was defined as histopathological evidence of inflammatory changes in biopsy specimens obtained from the gastrointestinal tract, including chronic inflammatory infiltrates, microscopic colitis, gastritis, ileitis, or other documented inflammatory histological changes. Patients were classified according to the final histopathological report as having either no microscopic inflammation or biopsy-proven microscopic inflammation. Histopathological findings were used to define the study outcome, whereas endoscopic appearance was used to identify patients with no visible mucosal abnormality.

Because the study included both upper and lower gastrointestinal procedures, the outcome was defined at the participant level according to the presence or absence of biopsy-proven microscopic inflammation in any sampled gastrointestinal site. The analysis was not intended to distinguish the diagnostic performance of the inflammatory indices across individual histopathological subtypes or anatomical sites.

Study Grouping

The participants were divided into two groups based on the endoscopic and histopathological findings. Patients who had no evidence of intestinal inflammation on endoscopy or biopsy were grouped into group A (normal endoscopy and biopsy). Group B consisted of patients with normal endoscopic findings but biopsy-proven microscopic inflammation.

Data analysis

All collected data were recorded and analyzed using the Statistical Package for the Social Sciences (SPSS) version 26 (IBM Corp, Armonk, NY). The quantitative variables, such as age, neutrophil count, lymphocyte count, platelet count, SII, NLR, and PLR, were examined for normality and reported as mean±standard deviation or median (IQR), as appropriate. Qualitative variables like sex, smoking history, gastrointestinal symptom categories, history of endoscopy and biopsy, and biopsy results were presented as frequencies and percentages. To assess the differences in inflammatory ratios by CBC, Group A (normal endoscopy with no histopathological evidence of inflammation) was compared with Group B (normal endoscopy with biopsy-proven microscopic inflammation). The distribution of the inflammatory markers was not normal; therefore, the Mann-Whitney U test was used to compare NLR, PLR, and SII between the two groups. The relationship between the gastrointestinal symptom categories and prior endoscopy status was analyzed using the chi-square test. The independent associations of inflammatory indices and selected clinical variables with biopsy-proven microscopic gut inflammation were evaluated using binary logistic regression analysis. For logistic regression analysis, the binary outcome was coded as 0 for Group A (normal endoscopy with normal biopsy) and 1 for Group B (normal endoscopy with biopsy-proven microscopic inflammation). Therefore, an odds ratio greater than 1 indicates higher odds of belonging to Group B per one-unit increase in the corresponding predictor, whereas an odds ratio less than 1 indicates lower odds of belonging to Group B. NLR, PLR, and SII were evaluated as CBC-derived inflammatory indices, and their mathematical relationship arising from their shared neutrophil, platelet, and lymphocyte components was considered when interpreting the regression estimates. Variance inflation factor (VIF) statistics were assessed before regression analysis to evaluate potential multicollinearity. Odds ratios (ORs) and 95% confidence intervals (CIs) were determined. Statistically significant was defined as a p-value <0.05.

Ethical considerations

The study was approved by the institution's Ethical Review Committee of Liaquat University of Medical and Health Sciences, Jamshoro (LUMH/IRB/BE-1-C301/2026) before it was conducted. Before enrolment, all participants signed informed consent forms. The purpose and procedure of the study were communicated to participants, and the confidentiality of all collected data was maintained. No personal identifiers were provided or shared, and all data were used for research purposes.

## Results

Table 2 summarizes the demographic and clinical characteristics of the 350 participants. The majority of participants were aged 31-45 years (N=106, 30.3%), followed by 46-60 years (N=96, 27.4%). The participants were made up of 180 men (51.4%) and 170 women (48.6%). Of the total number of participants, 178 (50.9%) were smokers. The most common gastrointestinal symptom was blood in stool (N=73, 20.9%). A large proportion of participants (N=120, 34.3%) had symptoms that persisted for more than six months. Previous gastrointestinal disease was reported by 210 participants (60.0%), and 178 participants (50.9%) had a family history of gastrointestinal disease. Among the 350 participants, 106 (30.3%) had normal endoscopic findings with no histopathological evidence of inflammation (Group A), whereas 244 (69.7%) had normal endoscopic findings but biopsy-proven microscopic inflammation (Group B).

**Table 2:** Demographic characteristics of participants (N=350) Note. N=number of participants; f=frequency, %=percentage

Variable	f	%
Age	-	-
18-30 years	81	23.1
31-45 years	106	30.3
46-60 years	96	27.4
>60 years	67	19.1
Gender	-	-
Male	180	51.4
Female	170	48.6
Smoking status	-	-
Smoker	178	50.9
Non-smoker	172	49.1
Main gastrointestinal symptoms	-	-
Abdominal pain	72	20.6
Diarrhea	70	20.0
Constipation	71	20.3
Bloating	64	18.3
Blood in stool	73	20.9
Duration of gastrointestinal symptoms	-	-
<1 month	114	32.6
1-6 months	116	33.1
>6 months	120	34.3
Family history of gastrointestinal disease	-	-
Yes	178	50.9
No	172	49.1
Previous gastrointestinal disease	-	-
Yes	210	60.0
No	140	40.0
Prior endoscopy	-	-
Yes	172	49.1
No	178	50.9
Presence of microscopic gut inflammation	-	-
Group A (Normal endoscopy+normal biopsy)	106	30.3
Group B (Normal endoscopy+positive biopsy inflammation)	244	69.7

Table 3 compares CBC-derived inflammatory ratios between participants with normal biopsy findings (Group A) and those with positive biopsy inflammation despite a normal endoscopy (Group B). The mean ranks for NLR (204.40 vs. 108.97), PLR (206.28 vs. 104.64), and SII (211.94 vs. 91.61) were significantly higher in Group B as compared to Group A as determined by Mann-Whitney U testing (p<0.001 for all).

**Table 3:** Comparison of CBC-derived inflammatory ratios (NLR, PLR, SII) between participants with normal endoscopy and absent biopsy inflammation (Group A) and participants with normal endoscopy and positive biopsy inflammation (Group B) using Mann-Whitney U tests (N=350) Note. N=350 (Normal endoscopy+normal biopsy=106; Normal endoscopy+positive biopsy inflammation=244); Mann-Whitney U test was used for all comparisons; p values marked with *** indicate statistical significance at p<0.001.

Groups		N	Median (IQR)	Mean Rank	Sum of Ranks	U	Z	p
NLR	Group A (Normal endoscopy+normal biopsy)	106	2.10 (1.60-2.80)	108.97	11550.5	-	-	-
	Group B (Normal endoscopy+positive biopsy inflammation)	244	4.30 (3.10-5.50)	204.4	49874.5	5879.5	-8.108	<0.001***
PLR	Group A (Normal endoscopy+normal biopsy)	106	120 (90-160)	104.64	11092	-	-	-
	Group B (Normal endoscopy+positive biopsy inflammation)	244	210 (170-260)	206.28	50333	5421	-8.636	<0.001***
SII	Group A (Normal endoscopy+normal biopsy)	106	450 (320-610)	91.61	9711	-	-	-
	Group B (Normal endoscopy+positive biopsy inflammation)	244	950 (740-1,250)	211.94	51714	4040	-10.223	<0.001***

Table 4 presents the binary logistic regression model assessing the association between inflammatory indices, clinical characteristics, and biopsy-proven microscopic inflammation. NLR (OR=1.57, 95% CI: 1.340-1.835, p<0.001), PLR (OR=1.012, 95% CI: 1.004-1.020, p=0.003), and SII (OR=1.025, 95% CI: 1.019-1.031, p<0.001) were significantly associated with biopsy-proven microscopic inflammation. Of these, SII had the largest association with group membership. Age (p=0.899), gender (p=0.882), smoking (p=0.952), family history of gastrointestinal disease (p=0.930), and previous endoscopy (p=0.953) were not significantly associated with the outcome in the model.

**Table 4:** Binary Logistic Regression Analysis of the Association Between Inflammatory Indices, Clinical Characteristics, and Biopsy-Proven Microscopic Gut Inflammation (N = 350) Note: Model Performance (Nagelkerke R²=0.45; Hosmer-Lemeshow Test p-value=0.25; Classification Accuracy = 78%); VIF values ranged from 1.1 to 2.3 and were below the acceptable threshold of 5, indicating absence of significant multicollinearity; The binary dependent variable was coded as 0=Group A (normal endoscopy with normal biopsy) and 1=Group B (normal endoscopy with biopsy-proven microscopic inflammation), with Group B representing the modeled event. Accordingly, OR >1 indicates increased odds of membership in Group B, whereas OR <1 indicates decreased odds of membership in Group B. NLR, PLR, and SII were entered simultaneously into the multivariable model. Because these indices share components of the complete blood count and are mathematically related, multicollinearity was assessed using variance inflation factors. VIF values ranged from 1.1 to 2.3, providing no evidence of severe linear multicollinearity using a threshold of 5. However, the mathematical relationship among NLR, PLR, and SII was considered when interpreting their mutually adjusted coefficients. B=Unstandardized regression coefficient; SE=standard error; OR=odds ratio; CI=confidence interval; NLR=neutrophil-to-lymphocyte ratio; PLR=platelet-to-lymphocyte ratio; SII=systemic immune-inflammation index. Statistical significance was defined as *p<.05, **p<.01, and ***p<.001.

Model	B	SE	Wald	df	OR	95% CI for OR	p
NLR	0.450	0.080	31.64	1	1.57	1.340–1.835	<0.001^***^
PLR	0.012	0.004	9.00	1	1.012	1.004–1.020	0.003^**^
SII	0.025	0.003	69.44	1	1.025	1.019–1.031	<0.001^***^
Age	0.021	0.016	1.721	1	1.021	0.990–1.054	0.899
Gender	-0.050	0.338	0.022	1	0.951	0.490–1.845	0.882
Smoking status	0.020	0.334	0.003	1	1.020	0.530–1.963	0.952
Family history of gastrointestinal disease	-0.030	0.341	0.008	1	0.971	0.497–1.893	0.930
Prior endoscopy	-0.020	0.340	0.003	1	0.980	0.503–1.908	0.953
Constant (Group A vs. Group B)	-0.459	1.136	0.164	1	0.632	0.068–5.857	0.686

Table 5 shows that the association between the main gastrointestinal symptoms and prior endoscopy status was statistically significant (χ²=47.49, df=4, p<0.001). For participants who had previously undergone endoscopy, the most prevalent symptoms were abdominal pain (N=50) and diarrhea (N=45). In contrast, for participants who had not previously had endoscopy, blood in stool (N=56) and bloating (N=44) were the most common symptoms. The findings in this group indicate a significant difference in gastrointestinal symptom rates between those with and without a prior endoscopy.

**Table 5:** Association between prior endoscopy and main gastrointestinal symptoms among participants using chi-square test (N=350) Note: Chi-square test examined the relationship between prior endoscopy (Yes/No) and the main gastrointestinal symptom categories: abdominal pain, diarrhea, constipation, bloating, and blood in stool. χ²=47.49, df=4, p<0.001***, indicating a significant association.

Prior endoscopy	Abdominal pain	Diarrhea	Constipation	Bloating	Blood in stool	Total	x^2^(df=4)	p
Yes	50	45	40	20	17	172	-	-
No	22	25	31	44	56	178	-	-
Total	72	70	71	64	73	350	47.49	<0.001^***^

Table 6 shows that all three inflammatory markers-NLR, PLR, and SII-demonstrated statistically significant discriminatory performance for biopsy-confirmed inflammation among patients with normal endoscopic findings (all p<0.001). SII showed the best diagnostic performance, with the highest AUC (0.776, 95% CI: 0.720-0.831) and Youden Index (0.502), indicating the greatest ability among the three markers to discriminate patients with underlying histopathological inflammation despite normal endoscopic findings. PLR also demonstrated good discriminatory ability (AUC=0.729, 95% CI: 0.668-0.790; J=0.419), whereas NLR showed comparatively lower but statistically significant discriminatory ability (AUC=0.684, 95% CI: 0.620-0.748; J=0.332). Overall, SII demonstrated the strongest diagnostic performance for identifying biopsy-confirmed inflammation among patients with normal endoscopy.

**Table 6:** ROC Curve Analysis and Diagnostic Performance of NLR, PLR, and SII for Identifying Biopsy-Proven Microscopic Inflammation Among Patients with Normal Endoscopy (Group B) Note: ROC=receiver operating characteristic; AUC=area under the curve; SE=standard error; CI=confidence interval; NLR=neutrophil-to-lymphocyte ratio; PLR=platelet-to-lymphocyte ratio; SII=systemic immune-inflammation index; PPV = positive predictive value; NPV=negative predictive value. The optimal cutoff was defined as the threshold maximizing the Youden index (J=sensitivity+specificity - 1). Ninety-five percent confidence intervals for sensitivity, specificity, PPV, and NPV were estimated using the Wilson binomial method.

Marker	AUC (95% CI)	SE	p-value	Optimal cutoff*	Sensitivity, % (95% CI)	Specificity, % (95% CI)	PPV, % (95% CI)	NPV, % (95% CI)	Youden Index (J)
NLR	0.684 (0.620-0.748)	0.033	<0.001	≥3.30	64.3 (58.2-70.1)	68.9 (59.5-76.9)	82.6 (76.6-87.4)	45.6 (38.1-53.4)	0.332
PLR	0.729 (0.668-0.790)	0.031	<0.001	≥175	69.3 (63.2-74.7)	72.6 (63.5-80.2)	85.4 (79.8-89.6)	50.7 (42.8-58.5)	0.419
SII	0.776 (0.720-0.831)	0.028	<0.001	≥700	73.8 (67.9-78.9)	76.4 (67.5-83.5)	87.8 (82.6-91.6)	55.9 (47.7-63.7)	0.502

## Discussion

The present study evaluated the diagnostic and discriminatory performance of CBC-derived inflammatory ratios, including NLR, PLR, and SII, in identifying biopsy-proven microscopic gut inflammation despite normal endoscopic findings. All three inflammatory indices were significantly elevated among participants with microscopic inflammation on biopsy despite a normal endoscopic appearance. These findings are consistent with previous studies suggesting that CBC-derived inflammatory markers may serve as potential non-invasive indicators of inflammatory activity. Earlier studies have also reported associations between these markers and inflammatory conditions, particularly established inflammatory bowel disease [5,7]. Among the evaluated markers, SII showed the strongest association with microscopic gut inflammation and the highest discriminatory performance. Similar findings have been reported in studies demonstrating associations between higher SII levels and histological and endoscopic inflammatory activity in patients with ulcerative colitis [11,12].

The higher NLR, PLR, and SII observed among participants with microscopic inflammation despite normal endoscopic findings suggest that systemic inflammatory alterations may be detectable when histopathological abnormalities are not apparent endoscopically [13]. Clinically, these findings mean some patients with gastrointestinal symptoms may have normal-appearing mucosa despite biopsy-proven microscopic inflammation. In this context, endoscopic assessment alone may not fully identify histopathological abnormalities [14]. CBC-derived inflammatory ratios may therefore provide readily available adjunctive information for identifying patients who may have biopsy-proven microscopic inflammation despite a normal endoscopic appearance. However, these indices should be considered adjunctive markers and should not replace endoscopic or histopathological assessment.

These findings are similar to previous studies showing correlations between NLR, PLR, and inflammatory and autoimmune diseases [12]. Previous studies found higher inflammatory ratios in inflammatory bowel disease, autoimmune diseases, and chronic inflammatory diseases, suggesting immune activation and dysregulation throughout the body in inflammatory conditions [10,15]. Most previous studies, however, were conducted in patients who had already presented with clinical and endoscopic changes, demonstrating the presence of inflammatory disease [11]. The present study examined whether inflammatory indices were associated with biopsy-proven microscopic inflammation despite a normal-appearing endoscopic mucosa. This supports the potential contribution of these readily available inflammatory indices as non-invasive adjunctive biomarkers.

SII showed the strongest association and the highest discriminatory performance among the evaluated indices. This aligns with previous studies showing that SII was better than NLR and PLR at discriminating disease activity and clinical outcomes in UC patients [16]. SII also includes counts of neutrophils, platelets, and lymphocytes within the same time window and may therefore provide a more complete picture of inflammatory burden than individual ratios. Neutrophilia and thrombocytosis indicate inflammatory activation. Conversely, decreased lymphocytes have been associated with immune dysfunction and chronic inflammatory stress, highlighting the contribution of both innate and adaptive immune responses in chronic inflammation [17,18]. Overall, the assessment provided by SII might thus reflect its higher diagnostic/discriminatory performance for biopsy-proven microscopic gut inflammation. These findings align with previous studies showing that SII results may outperform traditional inflammatory and immune markers in inflammatory and immune-mediated diseases [16].

Logistic regression also demonstrated that, after adjusting for demographic and clinical parameters, NLR, PLR, and SII were independently associated with biopsy-proven microscopic inflammation. In contrast, age, gender, smoking, family history of gastrointestinal disease, and previous gastrointestinal endoscopy were not significantly associated with the outcome in the regression model. Similar to prior research, NLR, PLR, and SII were independently associated with biopsy-proven microscopic inflammation, indicating stronger statistical associations with this outcome in this sample. The selected demographic and clinical parameters showed weaker associations with the outcome than the inflammatory indices [19-21]. These results indicate that inflammatory ratios calculated from the CBC may provide useful adjunctive information for identifying biopsy-proven microscopic gut inflammation rather than the conventional clinical parameters. Moreover, the results show that inflammatory activation can occur even without some baseline demographic factors.

The association of gastrointestinal symptoms with a history of endoscopy in this study is also clinically relevant. Symptoms such as abdominal pain or diarrhea were more common among those who had undergone endoscopy previously. Conversely, bloating and fecal blood were more common among those with no prior endoscopic evaluation. Direct evidence for this pattern remains limited. Still, past research has shown that the gastrointestinal system is often evaluated because of chronic diarrhea, abdominal pain, alteration of bowel habits, and gastrointestinal bleeding. The results indicated that the clinical picture could affect the referral for endoscopic evaluation or the acceptance of health-care-seeking behavior [22,23]. This may reflect variations in symptomatology, referral patterns, or climatic factors affecting access to health care. Alternatively, these patients may have undergone repeated endoscopic evaluations without obvious inflammatory changes of the mucosa, highlighting the potential value of non-invasive adjunctive markers for identifying possible microscopic inflammation.

The results obtained in this study have significant therapeutic implications. Inflammatory ratios obtained from the CBC are easy to perform, inexpensive, and are routinely done in health care facilities. These may serve as adjunctive screening indices to determine the need for more regular follow-up, earlier endoscopic evaluation with biopsy, or additional evaluation when endoscopy is not available. These CBC-derived indices should be considered adjunctive markers and should not replace endoscopic or histopathological assessment. These inflammatory indices may provide a convenient, inexpensive, and useful adjunctive approach in low-resource settings where more sophisticated diagnostic tests are not always available to identify patients with possible intestinal inflammation.

Limitations

The results of this study should be interpreted with caution. First, the cross-sectional design does not permit causal inference between inflammatory ratios and the incidence of intestinal inflammation over time. Longitudinal studies are warranted to assess if increased inflammatory markers are associated with the progression of gastrointestinal disease. Second, the study was conducted in selected tertiary-care centers among symptomatic patients referred for endoscopic evaluation, which may limit the generalizability of the findings to primary-care settings, community populations, asymptomatic individuals, and patients with different referral patterns. Additionally, the study was conducted within the available institutional resources and funding framework, which may limit the feasibility and generalizability of the findings to institutions with different levels of diagnostic resources, infrastructure, and financial support. Third, although we used consecutive recruitment during the study period, the sampling was non-probability based, so selection bias cannot be excluded. Furthermore, although we addressed several important potential confounders through predefined exclusion criteria and adjustment for selected clinical and demographic variables, we cannot completely rule out residual confounding. In particular, subclinical inflammatory or infectious conditions not detected at recruitment could have affected CBC-derived inflammatory indices. Another limitation is that the study relied on single-time-point measurements of inflammatory markers rather than repeated assessments. Because inflammatory markers were measured at a single time point, we could not assess their temporal stability or reproducibility. The relatively high proportion of participants with biopsy-proven microscopic inflammation (244/350, 69.7%) should be interpreted in the context of the study population. Participants were symptomatic adults referred for gastrointestinal endoscopic evaluation and biopsy at tertiary-care centers rather than a community-based or screening population. Therefore, this proportion should not be interpreted as an estimate of the prevalence of microscopic gut inflammation in the general population. The sample's enrichment with symptomatic patients may also have increased the observed frequency of histopathological abnormalities. It may limit the applicability of the findings to asymptomatic individuals or lower-risk populations. Additionally, we did not analyze inflammatory markers in the context of specific histopathological subtypes of microscopic inflammation; this may have reduced the power to detect associations between inflammatory markers and biopsy findings.

## Conclusions

This cross-sectional study found that CBC-derived inflammatory indices, including NLR, PLR, and particularly SII, were significantly associated with biopsy-proven microscopic gut inflammation among patients with normal endoscopic findings. SII demonstrated the highest discriminatory performance among the evaluated indices. These findings suggest that CBC-derived inflammatory indices may be inexpensive, non-invasive adjunctive markers for identifying patients with histopathological inflammation despite a normal endoscopic appearance. However, because of the cross-sectional design and selected tertiary-care study population, these findings should be interpreted as evidence of association and diagnostic discrimination rather than temporal prediction or causation. Prospective studies in broader and more representative populations are needed to determine their clinical utility and generalizability.
